# Within-city spatial variations in PM_2.5_ magnetite nanoparticles and brain cancer incidence in Toronto and Montreal, Canada

**DOI:** 10.1038/s41598-024-58119-2

**Published:** 2024-05-27

**Authors:** Susannah Ripley, Barbara A. Maher, Marianne Hatzopoulou, Scott Weichenthal

**Affiliations:** 1 Department of Epidemiology, Biostatistics and Occupational Health, McGill University, Montreal, H3A 1G1 Canada; 2 Centre for Environmental Magnetism & Palaeomagnetism, Lancaster University, Lancaster, LA1 4YQ UK; 3 Department of Civil & Mineral Engineering, University of Toronto, Toronto, M5S 1A4 Canada

**Keywords:** Epidemiology, Cancer, Nanotoxicology

## Abstract

Magnetite nanoparticles are small, strongly magnetic iron oxide particles which are produced during high-temperature combustion and friction processes and form part of the outdoor air pollution mixture. These particles can translocate to the brain and have been found in human brain tissue. In this study, we estimated associations between within-city spatial variations in concentrations of magnetite nanoparticles in outdoor fine particulate matter (PM_2.5_) and brain cancer incidence. We performed a cohort study of 1.29 million participants in four cycles of the Canadian Census Health and Environment Cohort in Montreal and Toronto, Canada who were followed for malignant brain tumour (glioma) incidence. As a proxy for magnetite nanoparticle content, we measured the susceptibility of anhysteretic remanent magnetization (χ_ARM_) in PM_2.5_ samples (N = 124 in Montreal, N = 110 in Toronto), and values were assigned to residential locations. Stratified Cox proportional hazards models were used to estimate hazard ratios (per IQR change in volume-normalized χ_ARM_). ARM was not associated with brain tumour incidence (HR = 0.998, 95% CI 0.988, 1.009) after adjusting for relevant potential confounders. Although we found no evidence of an important relationship between within-city spatial variations in airborne magnetite nanoparticles and brain tumour incidence, further research is needed to evaluate this understudied exposure, and other measures of exposure to magnetite nanoparticles should be considered.

## Introduction

Outdoor air pollution, especially fine particulate matter (PM_2.5_), is among the leading causes of death and disease worldwide and is implicated in the development of numerous cancers^1^. Recently, research interest has turned to examining the effects of pollutants on the brain. A major hypothesized mechanism for the health effects associated with particulate matter exposures is the ability of inhaled particles to induce oxidative stress and an inflammatory response in the body^2^; inflammation has also been implicated in the development of brain cancer^3,4^. However, although there is a biologically plausible explanation for a relationship between exposures to particulate matter and brain cancer incidence, evidence in the literature is mixed. Although some studies found no association between PM_2.5_ and brain cancer incidence^5–7^, some components such as carbonaceous particles (components indicating a combustion source) in PM_2.5_ have been found to be positively associated with brain cancer incidence^8^. In addition, ultrafine particles (UFP, i.e., particles less than 100 nm in diameter) were associated with increased brain cancer incidence in a previous cohort study conducted in Toronto and Montreal, Canada^5^. Since there are few known modifiable risk factors for brain cancer, identifying and quantifying the effects of modifiable environmental exposures may be an important way to reduce brain cancer incidence.

While existing studies of outdoor air pollution and brain cancer generally focus on the most commonly measured pollutants such as mass concentrations of PM_2.5_, there is increased interest in novel air pollution exposure metrics that account for composition, toxicity, and/or size of particles. Specifically, measures that account for particle composition, toxicity, and size may vary at finer spatial scales than PM_2.5_ mass concentrations, which could make them more useful in epidemiologic studies of exposure variations within cities. One measure of interest is the magnetite nanoparticle content of outdoor PM_2.5_. Magnetite nanoparticles are small (< 100 nm in diameter), strongly magnetic iron oxide particles that are produced during high-temperature combustion and friction processes including both vehicle tailpipe emissions and brake-wear as well as industrial activity^9–11^. Moreover, existing evidence suggests that outdoor concentrations of magnetite nanoparticles measured in PM_2.5_ samples vary substantially within cities, much more so than traditional PM_2.5_ mass concentrations^12^.

The relationship between magnetite nanoparticles and brain cancer is of particular interest as these pollutants can enter the brain directly through the olfactory nerve, the neuroenteric system and via the circulation, and have been identified in human brains^13–15^. Once in the brain, magnetite may provoke redox activity that leads to oxidative stress^13^. In a study in Mexico City, neuroinflammatory markers were correlated with presence of metals in the frontal lobe in children and young adults^13,16^. The magnetite nanoparticles observed in human brains are co-associated with a range of other, potentially toxic, exogenous metal-bearing particles, including aluminum, titanium, nickel, and platinum^13,14^. Although urban populations are exposed to magnetite nanoparticles, and it is biologically plausible that such exposures could contribute to adverse health outcomes, to date there have been no epidemiologic studies of the health effects of exposure to magnetite nanoparticles in outdoor air pollution. The aim of this study was to estimate the association between within-city spatial variations in the concentration of magnetite nanoparticles, as represented by laboratory measurement of the anhysteretic remanent magnetization susceptibility (χ_ARM_) of outdoor PM_2.5_ (as described below) and incidence of brain cancer in Montreal and Toronto, Canada. As a secondary aim, we investigated whether associations between brain tumours and outdoor concentrations of nitrogen dioxide (a marker of the broader traffic-related air pollution mixture) and PM_2.5_ mass concentrations were modified by mass-normalized ARM susceptibility of PM_2.5_.

## Methods

### Cohort description

The Canadian Census Health and Environment Cohort (CanCHEC) is a population-based cohort that has been described previously^17,18^. The cohort includes multiple cycles of follow-up of Canadian Census records and includes non-institutionalized Canadians (aged 25 and older) who were among the approximately 20% of households selected for enumeration by the long-form Census questionnaire in one of the eligible census years^19^. These datasets were linked to postal code histories to obtain annual place of residence from Historical Tax Summary Files. CanCHEC includes information from Census questionnaires on individual-level and contextual variables including socioeconomic indicators, ethnicity, and place of residence, as well as environmental conditions^17^. Mortality data were linked from the Canadian Vital Statistics Death Database and cancer incidence data were linked from the Canadian Cancer Registry. The CanCHEC dataset was created under the authority of the Statistics Act and approved by the Executive Management Board at Statistics Canada (reference: 045-2015). This is equivalent to standard research ethics board approval. Informed consent was waived by the Executive Management Board at Statistics Canada because the database used in this study contains only deidentified individual records. All methods were carried out in accordance with relevant guidelines and regulations.

Our study population includes individuals in the 1991, 1996, 2001 or 2006 CanCHEC cohorts aged 25–90 years at baseline who lived in Toronto or Montreal for at least 2 years during follow-up. Since approximately 20% of households were randomly assigned to complete the long-form census in each cohort cycle, some individuals were enumerated on more than one long-form census. These individuals were assigned to the earliest cohort in which they appeared.

### Ascertainment of cancer diagnosis

**Figure 1:**
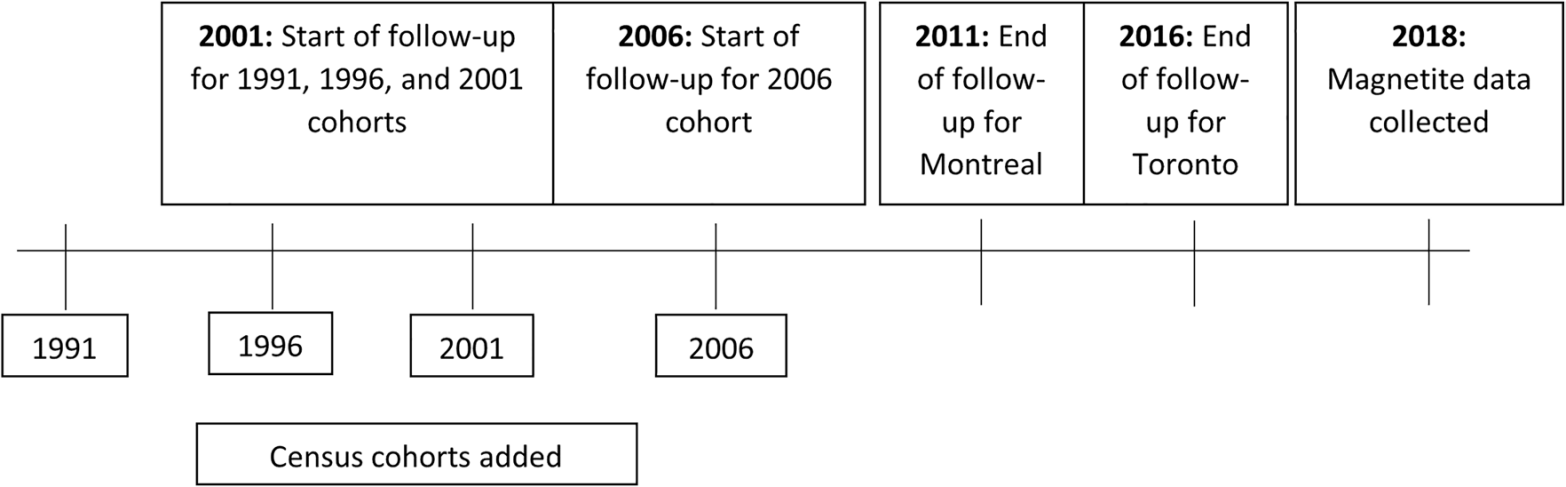
Study schema.

Cancer diagnoses in CanCHEC were identified using data linked to the CanCHEC cohorts from the Canadian Cancer Registry, a database that records incident primary cancers diagnosed for each person since 1992^20,21^. Participants were followed for first incidence of primary malignant brain tumour (defined by International Classification of Diseases, 10th Revision (ICD-10) codes C71.0–C71.9; corresponding to International Classification of Diseases for Oncology, 3rd revision [ICD-O-3] histologic codes for glioma M-938-M-948). Follow-up time started on Census day 2001 for the 1991, 1996 and 2001 cohorts, and Census day 2006 for the 2006 cohort. This restricted follow-up period was implemented to reduce potential error caused by extrapolating ARM susceptibility of PM_2.5_ (i.e., magnetite nanoparticle concentrations) many years into the past. For members of the study population living in Montreal, cases were only identified in the period 2001–2010 as cancer diagnosis data were not available in the province of Quebec for diagnosis years from 2011 onward. Participants were excluded if they had any cancer diagnosis in the 3 years prior to the start of follow-up to reduce the possibility of confounding by potential exposure to ionizing radiation in cancer treatment. The study schema is illustrated in Fig. 1.

### Spatial monitoring studies and estimation of magnetite nanoparticle exposures using anhysteretic remanent magnetization

PM_2.5_ samples were collected in outdoor monitoring campaigns conducted in 2018 in Montreal and Toronto, Canada. Monitoring sites were selected to capture important sources of ambient PM_2.5_ in each city while maximizing spatial coverage of the study area. A total of 124 sites in Montreal and 110 sites in Toronto were monitored. Mean daily temperatures over the sampled period ranged from 14.4 to 23.7 °C (57.9–74.7°F) in Montreal and 19.8–26.6 °C (67.6–79.9°F) in Toronto. Integrated 2-week PM_2.5_ samples were collected using Teflon filters and preset timers with a mix of Ultrasonic Personal Air Sample (UPAS) monitors (Access Sensor Technologies, Fort Collins, CO) at a flow rate of 1 L/min and cascade impactors at a flow rate of 5 L/min.

In order to quantify the content of magnetite particles in PM_2.5_ samples, anhysteretic remanent magnetization (ARM) was measured. ARM is roughly proportional to the concentration of ferrimagnetic minerals within a sample^22^ and specifically responds to the presence of magnetic nanoparticles with diameters between 30 and 50 nm^23,24^. First, PM_2.5_ samples (on PTFE filters) were exposed to four different direct current (DC) biasing fields of 0.06 mT, 0.08 mT, 1.0 mT and 1.2 mT. Subsequently, a 2G RAPID cryogenic magnetometer (2G Enterprises, Mountain View, CA) was used to measure the magnetic response of the samples. ARM measurements were also made of 20 blank PTFE filters and the mean of the ARM measurements taken on these blanks was subtracted from sampled filter values. ARM was expressed as a susceptibility of ARM normalized by the direct current (DC) field, calculated as the slope of the ARM(DC field) linear function. ARM susceptibility values were then normalized by air sampled volume (expressed as K_ARM_, a dimensionless quantity) for the primary analysis and by particulate mass (expressed as χ_ARM_, in units of m^3^/kg) in secondary analyses.

### ***Outdoor PM***_***2.5***_*** and nitrogen dioxide concentrations***

To evaluate the effects of spatial variations in other co-occurring pollutants, we assigned long-term estimates of outdoor PM_2.5_ and nitrogen dioxide (NO_2_) concentrations at residential address to cohort members in the same manner. Annual average outdoor PM_2.5_ mass concentrations were estimated using models described in detail previously^25^. Briefly, PM_2.5_ concentrations were estimated at a 1 × 1 km resolution using aerosol optical depth, a chemical transport model, and land-use data^25,26^. Annual average outdoor concentrations of NO_2_ were estimated using a land-use regression model^27^ in which estimates were derived from remote sensing and National Air Pollution Surveillance monitoring data; this model was developed from 2006 data and had a spatial resolution of 100 m^2^. PM_2.5_ and NO_2_ data indexed to DMTI Spatial Inc. postal codes were provided by CANUE (Canadian Urban Environmental Health Research Consortium).

### Exposure assignment

PM_2.5_ ARM susceptibility values, as well as PM_2.5_ and NO_2_ concentrations, were assigned directly to residential 6-digit postal codes (an area equivalent to approximately one city block face in urban areas) from the value at the closest measured site. Postal codes were linked to monitored points using latitude and longitude from the master postal code list (CanMap Postal Suite, version v2015.3, DMTI Spatial Inc., Markham). In cases where a single postal code was represented by multiple points of latitude and longitude, an average estimate for the postal code was created by equally weighting the multiple pollutant values across points. Median distance from monitors to postal code centroids was 916.5 m in Montreal and 1338.3 m in Toronto. Time-varying exposures were estimated using residential postal code histories from annual income tax filings, allowing for movement within and between cities. Exposures were assigned to cohort members at their residential address as 3-year moving averages with a 1-year lag (e.g., an individual’s exposure for 2008 was the mean of their exposures for 2005, 2006, and 2007). This is consistent with the standard exposure assignment used in many studies using the CanCHEC cohort since ambient PM_2.5_ is regulated in Canada based on a 3-year time window^28^. In addition, longer time windows (e.g., 10-years) would have required extrapolation of exposure estimates further back in time.

### Statistical analyses

Stratified Cox proportional hazards models were used to estimate hazard ratios describing the relationship between PM_2.5_ ARM susceptibility (volume-normalized, i.e., K_ARM_) and incidence of brain tumours. Follow-up time started with time of entry into the CanCHEC cohort (e.g., Census day 2001 for the 2001 cohort). Subjects were censored if they moved outside the cities of Montreal or Toronto, if they were lost to follow-up, at the end of study period, or at time of death, whichever came first. Data were accessed and analyzed in the secure facilities of the McGill-Concordia Research Data Centre located at McGill University. Statistical analyses were performed using SAS version 9.4 (SAS Institute, Cary, NC, USA) and R 4.2.2 (R Foundation for Statistical Computing, Vienna, Austria).

Variables used for regression adjustment were chosen based on a Directed Acyclic Graph (DAG) (Fig. S1). There are few well-established risk factors for primary brain tumours except for exposure to ionizing radiation and family history^29^. Nonetheless, we adjusted for a number of demographic and socioeconomic status variables which could confound the relationship through chance associations with the outcome. Specifically, we adjusted for age (5-year age groups as a strata variable), sex (male/female strata), immigration status (immigrant/nonimmigrant strata), Census cohort year (four categories as strata: 1991, 1996, 2001, and 2006), visible minority status, occupational level, educational attainment, marital status, and income quintile. Additionally, models were adjusted for PM_2.5_ mass concentrations and NO_2_ to evaluate the sensitivity of effect estimates to spatial variations in long-term exposures to these pollutants.

As an additional analysis, we examined the effects of PM_2.5_ and NO_2_ stratified by mass-normalized ARM susceptibility (χ_ARM_). When stratifying by ARM susceptibility, our goal in the estimation of NO_2_ effects on brain cancer incidence was to investigate whether the mixture of traffic-related air pollutants (of which NO_2_ is a marker) is more harmful in areas with greater ARM susceptibility; in the PM_2.5_ analysis, our goal was to evaluate whether the effect of exposure to fine particles was greater in areas where PM_2.5_ ARM susceptibility is higher. We performed Cox proportional hazards regression as described above and below the median of χ_ARM_ values.

### Sensitivity analyses

Some individual-level risk factor variables, notably cigarette smoking and body mass index (BMI), are not available in the CanCHEC database. Although the evidence linking smoking to brain cancer incidence is inconsistent, and some studies suggest no relationship^30^, cigarette smoking is an important cause of human cancer and meta-analysis suggests a possible association with brain cancer incidence^31^. Similarly, evidence suggests a possible assocation of obesity with some types of brain cancer^32^. Smoking and BMI are not causes of outdoor air pollutant concentrations, so they are not confounders in the standard definition^33^. Nonetheless, chance associations could confound the relationship between outdoor PM_2.5_, NO_2_ or ARM susceptibility and brain tumour incidence, and the indirect adjustment method was applied to address this possibility. The indirect adjustment method has been described in detail previously^34^; briefly, this method uses data on the correlation between measured covariates and unmeasured risk factors from a secondary data source, as well as estimates of the relationship between the missing risk factors and incidence of the outcome. We used data from multiple cycles of the Canadian Community Health Survey, a biannual national health survey that has the same target population as the Canadian census (i.e., the Canadian population) and collects data on health and lifestyle characteristics including smoking and BMI. The relationships between smoking and brain cancer, as well as BMI and brain cancer, were estimated from the literature based on systematic reviews and meta-analyses of the existing evidence^31,32^.

## Results

Cohort characteristics are presented in Table 1. In total, we identified approximately 1300 eligible cases of malignant primary brain tumours over 13.6 million person-years of follow-up in 1.29 million individuals (all numbers rounded to the nearest 100 to satisfy institutional confidentiality requirements). Incident brain tumours were identified at a higher rate in people of increased age, in men relative to women, and in people who identified as white relative to those who identified as visible minorities (Table 1).

**Table 1:** Descriptive statistics at baseline for the study cohort of people living in Toronto or Montreal (1991, 1996, 2001, and 2006 CanCHEC cohorts).

Characteristic	Person-Years	Participants	Incident brain tumours	Rate (Per Person-Year)
Total	13,636,200	1,291,900	1300	9.5
Sex				
Male	6,258,300	608,300	700	11.2
Female	7,377,900	683,600	600	8.1
Immigrant status				
Non-immigrant	7,032,800	710,400	700	10.0
Immigrant	6,603,400	581,500	600	9.1
City of residence				
Montreal	5,079,400	564,600	500	9.8
Toronto	8,556,800	727,300	800	9.3
Age group				
25–34	2,014,600	193,700	200	9.9
35–44	3,071,500	288,300	300	9.8
45–54	3,057,900	287,900	300	9.8
55–64	2,175,500	205,700	200	9.2
65–74	1,457,900	137,400	200	13.7
75–84	1,284,700	123,300	200	15.6
85–89	574,100	55,500	100	17.4
Occupational class				
Management	1,151,500	107,300	100	8.7
Professional	2,132,000	193,500	200	9.4
Skilled, technical & supervisory	2,513,700	233,600	200	8.0
Semiskilled	3,032,200	279,200	300	9.9
Unskilled	993,800	89,800	100	10.1
No occupation/not in labour force	3,812,900	388,500	500	13.1
Income quintile				
Lowest	2,727,300	260,900	200	7.3
Second lowest	2,727,200	274,300	300	11.0
Middle	2,722,800	261,000	300	11.0
Second highest	2,731,500	254,200	300	11.0
Highest	2,727,400	241,400	300	11.0
Educational attainment				
Less than high school graduation	3,725,400	351,700	400	10.7
High school graduation with/without trades certificate	4,053,500	382,700	400	9.9
Some postsecondary or college diploma	2,405,900	238,000	200	8.3
University degree	3,451,400	319,500	300	8.7
Cohort				
1991	3,778,500	337,000	400	10.6
1996	5,225,900	465,700	600	11.5
2001	3,269,000	283,300	200	6.1
2006	1,362,800	205,900	100	7.3
Marital status				
Single	2,695,600	263,600	200	7.4
Common-law	929,600	101,000	100	10.8
Married	8,038,100	723,400	900	11.2
Separated	401,000	38,900	NA	NA
Divorced	849,000	84,300	100	11.8
Widowed	722,900	80,600	100	13.8
Visible minority status				
Not defined as visible minority	9,932,000	962,300	1100	11.1
Visible minority	3,704,00	329,600	200	5.4

All numbers are rounded to the nearest 100 for confidentiality and may not add up to the total; NA denotes counts below 100 which are suppressed for confidentiality.

The mean volume-normalized ARM susceptibility (K_ARM_) across all eligible person-years was 5.8 × 10^−14^ (SD = 3.4 × 10^−14^) and mean mass-normalized ARM susceptibility (χ_ARM_) was 9.3 × 10^−6^ m^3^/kg (SD = 6.7 × 10^−6^ m^3^/kg). Spatial variations in ARM susceptibility were much greater than spatial variations in PM_2.5_ mass concentrations. The mean PM_2.5_ concentration was 9.4 μg/m^3^ (SD = 1.3 μg/m^3^) and the mean NO_2_ concentration was 21.2 ppb (SD = 5.5 ppb) (Table 2). ARM susceptibility parameters showed little correlation with PM_2.5_ mass concentration or NO_2_ concentration. Spatial variations in K_ARM_ (volume-normalized) were very weakly correlated with PM_2.5_ mass concentration (r = − 0.0007) and NO_2_ (r = 0.0496), and similarly spatial variations in χ_ARM_ (mass-normalized) were very weakly correlated with PM_2.5_ (r = 0.023) and NO_2_ (r = − 0.028). Distributions of PM_2.5_ and NO_2_ at measured sites were similar to values across the entire study area (Table S1).

**Table 2:** Descriptive statistics for ambient pollutant concentrations and PM_2.5_ ARM susceptibility characteristics across all person-years.

Characteristic	Mean (SD)	Median	IQR	Percentile			
				1st	25th	75th	99th
Pollutant concentrations							
PM_2.5_ (µg/m^3^)	9.4 (1.3)	9.5	1.6	6.9	8.5	10.1	13
NO_2_ (ppb)	21.2 (5.5)	21.0	7.5	10.4	17.1	24.6	36.4
ARM susceptibility parameters							
K_ARM,_ volume specific (× 10^−14^, unitless)	5.8 (3.4)	4.9	3.1	0.1	4.0	7.1	18.0
* χ*_ARM,_ mass specific (× 10^−6^ m^3^/kg)	9.3 (6.7)	7.0	4.0	2.0	6.0	10.0	39.0

ARM: anhysteretic remanent magnetization; IQR: interquartile range; NO_2_: nitrogen dioxide; PM_2.5_: fine particulate matter; SD: standard deviation.

Cox regression model results are presented in Table 3. Models showed no association between volume-normalized ARM susceptibility (K_ARM_) and brain cancer incidence (HR per 3.0 × 10^−14^: 0.998, 95% CI 0.988, 1.009). Long-term exposures to PM_2.5_ were inversely though nonsignificantly associated with brain cancer incidence (HR per 3 µg/m^3^: 0.833, 95% CI 0.681, 1.021). When stratified by mass-normalized ARM susceptibility (χ_ARM_), the effect of PM_2.5_ was closer to the null (i.e., indicating a less strong protective effect) above the median X_ARM_ (HR: 0.899, 95% CI 0.774, 1.043) relative to below the median (HR: 0.711, 95% CI 0.374, 1.350), but estimates were imprecise. Similarly, long-term average exposures to NO_2_ were not associated with brain cancer incidence (HR per 10 ppb: 0.963, 95% CI 0.876, 1.058). In stratified analyses, the point estimate of the effect of NO_2_ above the median of χ_ARM_ (HR: 0.945, 95% CI 0.848, 1.053) was protective whereas below the median of χ_ARM_ the effect was deleterious (HR: 1.020, 95% CI 0.877, 1.195); however, confidence intervals were wide and overlapping. For all pollutants, indirect adjustment for smoking and body mass index had little effect on hazard ratios (Table 3).

**Table 3:** Crude and indirectly adjustment hazard ratios for incident primary malignant brain tumours.

Pollutant	Crude HR (95% CI)	Indirectly adjusted HR (95% CI)
PM_2.5_ (per 3 µg/m^3^)		
Overall	0.845 (0.692, 1.031)	0.833 (0.681, 1.021)
Below median ARM susceptibility (χ_ARM_)	0.736 (0.387, 0.898)	0.711 (0.374, 1.350)
Above median ARM susceptibility (χ_ARM_)	0.895 (0.771, 1.092)	0.899 (0.774, 1.043)
NO_2_ (per 10 ppb)		
Overall	0.965 (0.881, 1.057)	0.963 (0.877, 1.058)
Below median ARM susceptibility (χ_ARM_)	1.037 (0.891, 1.206)	1.020 (0.877, 1.195)
Above median ARM susceptibility (χ_ARM_)	0.933 (0.841, 1.036)	0.945 (0.848, 1.053)
ARM susceptibility normalized by volume (K_ARM_) (per 3.0 × 10^−14^)	1.000 (0.894, 1.118)	0.998 (0.988, 1.009)

K_ARM_: volume-normalized anhysteretic remanent magnetization susceptibility of PM_2.5_; X_ARM_: mass-normalized anhysteretic remanent magnetization susceptibility of PM_2.5._

## Discussion

We conducted a population-based cohort study examining the relationship between within-city spatial variations in fine particle (PM_2.5_) ARM susceptibility on malignant brain tumour incidence in two Canadian cities. We found no relationship between exposures to volume-normalized ARM susceptibility of PM_2.5_ and brain tumour incidence; moreover, we found no evidence that the effects of other long-term outdoor pollutant exposures (i.e., PM_2.5_ mass concentration and NO_2_ as a marker for traffic-related air pollution) were modified by the mass-normalized ARM susceptibility of fine particles. In general, our findings do not support a relationship between spatial variations in ARM susceptibility of PM_2.5_, a measure which corresponds to the presence of magnetite nanoparticles (~ 30–50 nm diameter), and incidence of brain cancer. We identified a non-significant inverse effect of PM_2.5_ on brain cancer incidence which we cannot explain. However, this result is similar to protective effects previously observed for the effect of PM_2.5_ on brain cancer incidence, such as by Jorgenson et al. (HR per 3 µg/m^3^: 0.985, 95% CI 0.635, 1.54)^35^, by Harbo Poulsen et al. (OR per 3 µg/m^3^: 0.992, 95% CI 0.946, 1.039)^8^, or by Weichenthal et al. (HR per 3 µg/m^3^: 0.907, 95% CI 0.762, 1.079)^5^.

Although we found no effect of PM_2.5_ ARM susceptibility on brain cancer incidence, nonetheless the health effects of exposures to magnetite nanoparticles merit further study with different exposure metrics. Exposure to magnetite particles in human cells in vitro can induce reactive oxygen species generation^36^, which contributes to the oxidative stress pathway that may be responsible for many of the observed adverse health effects of PM exposure. Further, experiments in rat cortical neurons suggest that exposure to magnetite may play a role in the development of neurodegenerative diseases such as Alzheimer’s disease^37^. Magnetite nanoparticles observed in the human brain are co-associated with other exogenous metal-bearing nanoparticles, including titanium, aluminum, platinum, nickel, and cobalt^13,14^. Toxicological studies have suggested that metal-rich ultrafine particles are able to access all major organs^38–41^, suggesting their relevance to health outcomes including those affecting the brain^42^. Given the toxicological evidence, there remains a need for future epidemiologic studies assessing the effects on health of magnetite nanoparticle exposures.

Future studies may benefit from exploring different measures of magnetite nanoparticles. We used the room temperature ARM of PM_2.5_ as a surrogate measure of magnetic nanoparticle content as it reflects the concentration of particles of approximately 30–50 nm in diameter^23,24^. However, it is possible that the ARM in PM_2.5_ samples may not be a good proxy for sampling the actual nanoparticle size range. We are most interested in the smallest particles (e.g., UFPs, which have diameter less than 0.1 µm) as there is evidence that they may be relevant to the development of brain cancer^5^. Given the potential relevance of UFPs to brain health, a possible direction for future research could be the measurement of magnetic parameters on the ultrafine fraction of particulate matter: for example, Gonet et al. analyzed isothermal remanent magnetization (IRM, a measure of magnetic remanence which results from short-term exposure to strong magnetizing fields) on size-fractionated particles sampled from brake-wear emissions (using 14 size fractions ranging from 0.016 to 10 µm)^43^. Although an increasing body of literature exists describing magnetic parameters of particles collected from air samples^44,45^ or from tree leaf surfaces near roadways^9,46–48^, it remains to be determined which particle magnetic properties and which particle size fractions are most relevant in health studies; future studies may consider size-resolved evaluation of magnetic characteristics of particles as a step towards assessment of their potential health impact. Additionally, a focus on low-temperature (LT) magnetic measurements may better characterize the UFP fraction. Muxworthy^44^ performed LT magnetic remanence measurements and found significantly higher concentrations of magnetite nanoparticles than previously estimated using room-temperature (RT) measures. In addition, Sheikh et al.^49^ collected air samples from the London Underground and analyzed them using ARM as well as both room temperature saturation isothermal remanence (RT-SIRM) and low temperature SIRM (LT-SIRM). Given evidence that the predominant size range of magnetite nanoparticles identified in the brain is 5–20 nm^50^, and that magnetite nanoparticles in this size range are more accurately quantified using LT methods^44^, future studies of PM may be better served by LT magnetic measurements rather than the RT measures performed here.

This study had several notable strengths, including high-resolution estimates of spatial variations in the ARM susceptibility of PM, the availability of updated exposure information for subjects moving within and between cities, and time-varying estimates of NO_2_ and PM_2.5_ exposures, as well as detailed individual-level data on potential confounders. A further advantage is the availability of data on incident, rather than prevalent brain tumour diagnoses. However, our study also had a number of limitations. First, the PM_2.5_ ARM susceptibility values were based on measurements of air filters collected during 2-week monitoring periods in 2018 (i.e., after the end of the follow-up period), and due to the absence of historical measurements it was not possible for us to extrapolate ARM susceptibility estimates backward in time. This is a source of possible exposure measurement error; however, a systematic difference in the degree of exposure error between brain cancer cases and non-cases is not expected and therefore bias would tend toward the null. Additionally, since major changes in spatial patterns of roadway infrastructure have not occurred during the study period, we did not expect major changes in the spatial patterns of tailpipe and brake wear emissions. Next, measurements of PM_2.5_ ARM susceptibility were made at room temperature, rather than at low temperature (e.g., liquid nitrogen, 77 K, or helium, 4.2 K, temperatures). Recent, low temperature-based studies show that both the total magnetite content and the numbers of ultrafine particles < 10 nm in size (magnetically ‘invisible’ at room temperature) are being routinely under-estimated in magnetic characterisation of particulate air pollution^49^. Further, we assume that the use of 2-week monitoring periods represents a sufficient approximation to long-term average spatial variations in PM_2.5_ ARM susceptibility. We based this assumption on existing evidence that suggests that the spatial pattern of pollutant concentrations derived from short-term monitoring campaigns remains relatively stable over time^51,52^. Although the ARM susceptibility measurements were collected after the end of follow-up, spatial contrasts are assumed to be representative of earlier spatial contrasts within each city during the follow-up period.

A second limitation was the absence of individual-level data on potential confounders such as smoking and body mass index. However, as described in the conceptual directed acyclic graph (Fig. S1), these individual-level variables are not likely causes of long-term air pollution exposures including PM_2.5_ ARM susceptibility, so they are not strictly confounders. Nonetheless, we performed an indirect adjustment method to account for confounding that could occur by chance associations between PM_2.5_ ARM susceptibility and individual-level variables. Similarly, we lacked individual-level data on other potential causes of brain cancer (e.g., family history of brain cancer or exposures to ionizing radiation). Since these factors were not present in the ancillary database we used to perform the indirect adjustment, we were unable to adjust for them. It is possible that potential confounding by these or other unmeasured confounders remains, if there exists a systematic relationship between the potential confounders and spatial variations in outdoor PM_2.5_, NO_2_ or ARM susceptibility. Lastly, the definition of the study outcome as all primary malignant brain tumours could potentially obscure any effect of ARM on individual tumour types as some tumour types may be more strongly related to ARM susceptibility than others. However, there is little evidence from previous studies to suggest which types of tumours may be more or less susceptible to the effects of inhaled pollutants. Although an examination of specific tumour subtypes may be an important opportunity for future research, given the relatively small number of events, we focused on the combined outcome definition to maximize precision.

In conclusion, we performed the first cohort study of spatial variations in ARM susceptibility of outdoor PM_2.5_ and incident brain tumours. We did not find an association between ARM susceptibility, a measure which is proportional to the concentration of magnetite nanoparticles, and brain cancer incidence. Nonetheless, future studies should continue to exposure explore the potential health impacts of magnetite nanoparticles using alternative exposure metrics due to the prevalence of exposure to these pollutants in urban areas.

### Supplementary Information

Supplementary Information.

## Data Availability

CanCHEC cohort data are held in secure Research Data Centres facilities managed by Statistics Canada. These can be accessed through the microdata access portal application process. The application process and procedures are available online here: www.statcan.gc.ca/en/microdata/data-centres/access.
