# Supplementary material for: Within-city spatial variations in PM2.5 magnetite nanoparticles and brain cancer incidence in Toronto and Montreal, Canada
Source: Sci Rep. 2024 May 27;14:12136. doi: 10.1038/s41598-024-58119-2 (PMC11130222; doi:10.1038/s41598-024-58119-2)
Supplement: Supplementary file 1 — Supplementary Information. [file 41598_2024_58119_MOESM1_ESM.docx]

Within-city spatial variations in PM_2.5_ magnetic properties and brain cancer incidence in Toronto and Montreal, Canada

Supplemental Materials

Susannah Ripley^1^, Barbara H. Maher,^2^ Marianne Hatzopoulou^3^, Scott Weichenthal^1^

^1^Department of Epidemiology, Biostatistics and Occupational Health, McGill University, Montreal, Canada, H3A 1G1

^2^Centre for Environmental Magnetism & Palaeomagnetism, Lancaster University, Lancaster, UK, LA1 4YW

^3^Department of Civil & Mineral Engineering, University of Toronto, Toronto, Canada, M5S 1A4

**Figure S1.** Directed acyclical graph for anhysteretic remanent magnetization (ARM) susceptibility of outdoor PM_2.5_ concentrations (a surrogate measure of magnetite nanoparticle concentrations) and brain tumour incidence. Parameters in grey are unmeasured variables, parameters in red are potential confounding factors, and parameters in blue are included as strata variables in the Cox proportional hazards regression models.


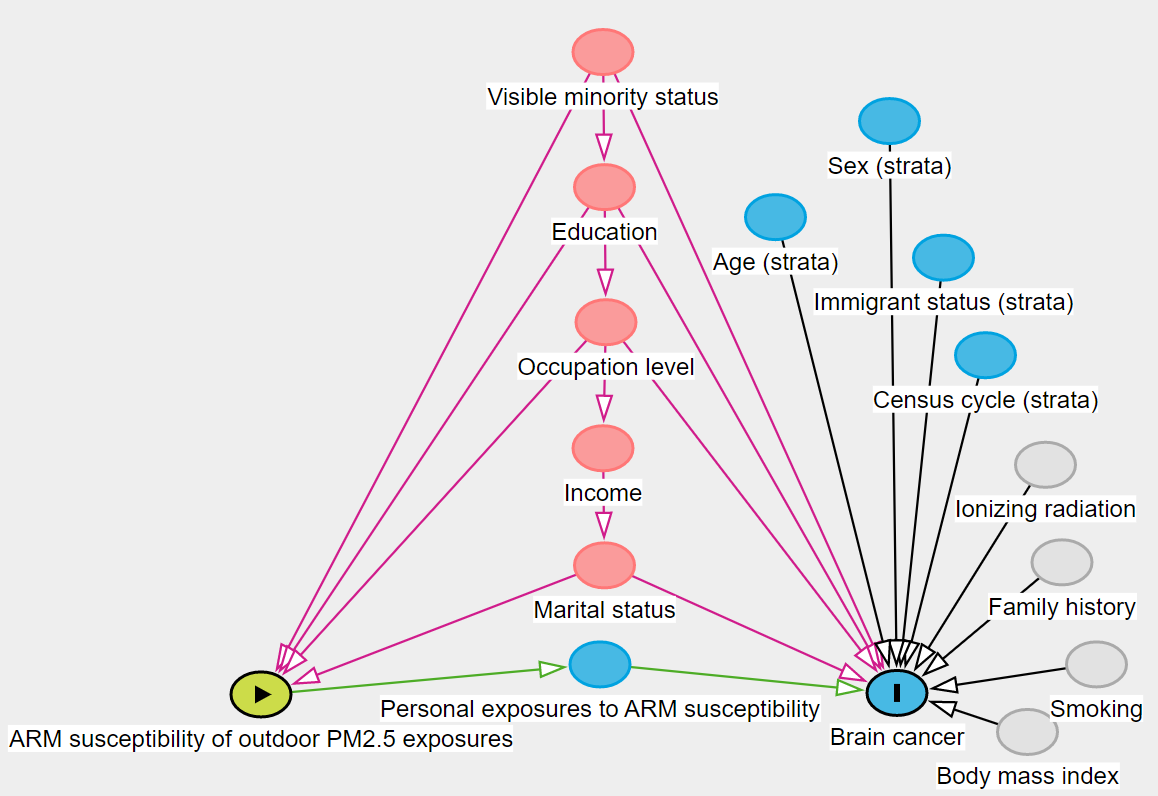


Table S1. Summary statistics for 2018 air pollutant variables at measured points and over the entire study area

|  | NO_2_ (ppb) | PM_2.5_ (µg/m^3^) |
| --- | --- | --- |
| Values at measured points (mean [median], Q1-Q3) | 22.8 [22.5], 20.7-24.8 | 7.7 [7.8], 7.5-8.0 |
| Values at all postal code points (mean [median], Q1-Q3) | 22.4 [22.3], 19.7-25.1 | 7.8 [7.9], 7.6-8.1 |
